# Factors Influencing How Providers Assess the Appropriateness of Video Visits: Interview Study With Primary and Specialty Health Care Providers

**DOI:** 10.2196/38826

**Published:** 2022-08-24

**Authors:** Caroline Gray, Charlie Wray, Rebecca Tisdale, Camila Chaudary, Cindie Slightam, Donna Zulman

**Affiliations:** 1 VA Palo Alto Healthcare System Menlo Park, CA United States; 2 Division of Hospital Medicine, San Francisco Veterans Affairs Medical Center San Francisco, CA United States

**Keywords:** virtual care, decision-making, qualitative, virtual visits, web-based, carer, video, telephone, telemedicine, appointments, caregiver

## Abstract

**Background:**

The rapid implementation of virtual care (ie, telephone or video-based clinic appointments) during the COVID-19 pandemic resulted in many providers offering virtual care with little or no formal training and without clinical guidelines and tools to assist with decision-making. As new guidelines for virtual care provision take shape, it is critical that they are informed by an in-depth understanding of how providers make decisions about virtual care in their clinical practices.

**Objective:**

In this paper, we sought to identify the most salient factors that influence how providers decide when to offer patients video appointments instead of or in conjunction with in-person care.

**Methods:**

We conducted semistructured interviews with 28 purposefully selected primary and specialty health care providers from the US Department of Veteran’s Affairs health care system. We used an inductive approach to identify factors that impact provider decision-making.

**Results:**

Qualitative analysis revealed distinct clinical, patient, and provider factors that influence provider decisions to initiate or continue with virtual visits. Clinical factors include patient acuity, the need for additional tests or labs, changes in patients’ health status, and whether the patient is new or has no recent visit. Patient factors include patients’ ability to articulate symptoms or needs, availability and accessibility of technology, preferences for or against virtual visits, and access to caregiver assistance. Provider factors include provider comfort with and acceptance of virtual technology as well as virtual physical exam skills and training.

**Conclusions:**

Providers within the US Department of Veterans Affairs health administration system consider a complex set of factors when deciding whether to offer or continue a video or telephone visit. These factors can inform the development and further refinement of decision tools, guides, and other policies to ensure that virtual care expands access to high-quality care.

## Introduction

The COVID-19 pandemic spurred rapid and widespread implementation of virtual care, including both video- and telephone-based visits, to address acute and chronic needs of patients. Many welcomed the availability of virtual care given benefits such as increased access to care, less travel time for patients, and often lower costs for both patients and health care systems [1]. Providers and patients have also reported unexpected advantages such as greater convenience and the ability to assess patients in their home environments [2].

While in-person care delivery has largely resumed, virtual care continues to play a major role in how health care systems deliver care to patients [3]. Because of this, identifying optimal approaches to virtual care delivery that ensure patient safety, satisfaction, quality of care, and equitable access will remain a critical challenge facing health care organizations [4,5]. Due to the rapid implementation of virtual care during the pandemic, many providers were asked to provide virtual care with little or no formal training and without clinical guidelines and tools to assist with decision-making. Currently, these guidelines and tools are beginning to take shape, and it is critical that they are informed by an in-depth understanding of *how* providers make decisions about virtual care in their clinical practices.

Currently, there is a plethora of qualitative studies describing provider and patient attitudes toward virtual care, as well as perceived barriers and facilitators to virtual care implementation and adoption [2,6-8]. Well-known barriers include lack of institutional support and the infrastructure to support the technology needed for virtual care, low levels of digital literacy among both patients and providers, and poor integration of virtual modalities into existing clinical workflows, to name a few [2,8,9]. Though these studies may improve uptake and implementation of virtual care, they sometimes lack specifics on how and when to provide virtual care as a substitute or adjunct to in-person care. Fewer studies have examined how non–mental health care providers more generally make decisions about when to use virtual modalities [10,11]. Systematic identification of the factors that providers consider when assessing the suitability of virtual care for a given patient and clinical need may inform the aforementioned tools and guidelines necessary for accessible, high-quality care.

The US Department of Veterans Affairs (VA) has been a leading health care organization in the use of virtual care modalities, even before the onset of the COVID-19 pandemic [12,13]. Through a qualitative assessment of VA providers from diverse clinical settings, we sought to identify the most salient factors that impact providers’ decisions about when to offer patients virtual care.

## Methods

### Participants and Study Design

As part of a study of VA’s implementation of virtual care and distribution of video-enabled tablets to veterans with access barriers, we conducted a qualitative study of a national sample of VA clinicians. We used a combination of administrative data and provider referral to purposefully sample participants. A majority of providers had only begun offering virtual care in the last 1 to 2 years at the time of interviews. Rather than focus on a single specialty, we strove for variation in our interview sample [14] and selected providers from the following 4 diverse areas of clinical practice: primary care, cardiology, spinal cord injury, and palliative care. With guidance from VA’s Office of Connected Care, these specialties were chosen due to higher use of virtual care. Additionally, they offer diverse types of services offered during a clinical visit. Through administrative data, we identified practitioners who had frequently used video visits in the previous calendar year relative to their peers in similar clinical practices. Providers were recruited from several US geographic regions—West, Midwest, South, and Northeast. The participants were sent messages to their institutional email addresses explaining the purpose of the study and asked if they would like to participate. A total of 26 physicians and 2 nurse practitioners agreed to participate in an interview, for a total of 28 providers. Participant characteristics are summarized in Table 1.

**Table 1:** Characteristics of health care providers (N=28).

Characteristics		Values, n (%)
**Clinical specialty or practice**		
	Primary care	11 (39)
	Cardiology	7 (25)
	Spinal cord injury	5 (18)
	Palliative care	5 (18)
**Gender**		
	Women	16 (57)
	Men	12 (43)
**Setting**		
	Rural	5 (18)
	Urban or suburban	23 (82)
**Years of practice**		
	Less than 5 years	4 (14)
	Over 5 years	24 (86)

### Ethics Approval

This quality improvement initiative was reviewed and designated as nonresearch by the supporting VA program office, the Stanford University Institutional Review Board, and VA Research Administration.

### Data Collection

Two researchers, a medical sociologist with expertise in qualitative methods (CG) and an internist with qualitative training (RT), conducted interviews with the providers to learn about their experiences offering virtual care prior to and during the COVID-19 pandemic. The interviews took place between December 2020 and June 2021, with each specialty interviewed consecutively to ensure greater consistency. The interview questions focused on circumstances under which providers choose to offer virtual care, preferences for virtual care or in-person care, and perceptions of scenarios where virtual care was inappropriate or less optimal. The providers were also asked to reflect on needed skills and training around virtual care and perceived barriers to providing virtual care more frequently. The interviews were conducted using Microsoft Teams, which lasted approximately 30 minutes, and with permission from interview participants, they were videorecorded and transcribed by a professional transcription service.

### Data Analysis

To identify the primary factors informing provider decision-making around virtual care, we employed a qualitative descriptive approach [15], using constant comparison [16,17] to further reduce and synthesize data. First, the research team inductively reviewed 5 transcripts and identified emergent codes, combined these codes with deductive codes derived from the interview questions, and created a codebook used to code all transcripts. The transcripts were uploaded into Atlas.ti (ATLAS.ti Scientific Software Development GmbH), a software that facilitates qualitative data analysis, and coded according to the codebook. After transcripts were coded, the codes and their associated text were reviewed collectively by the team and then grouped together into larger categories. During this process, we identified themes by assessing for repetition and emphasis of specific points. Finally, all team members participated in selecting exemplary quotes and sorting themes into 3 categories of factors that appeared to most impact provider decision-making.

## Results

### Clinical, Provider, and Patient Factors Impacting the Decision to Use Virtual Care

Thematic analysis revealed that provider decisions about whether to continue with or initiate a virtual visit is driven by clinical, patient, and provider factors (Textbox 1). Although we observed some variation related to specific aspects of the different clinical focus areas, the factors discussed here were noted across all 4 specialties.

**Thematic categorization of factors influencing provider decision-making.**

**Clinical factors**

- Patient acuity
- Need for additional tests or labs
- Change in patient’s status or overall stability
- First visits and patients with no record of recent medical examination

**Patient factors**

- Patient’s ability to articulate symptoms or needs
- Availability and accessibility of technology
- Preferences regarding virtual visits
- Access to caregiver assistance

**Provider factors**

- Comfort with and acceptance of virtual technology
- Knowledge about how to conduct physical exam and assessment virtually

### Clinical Factors Impacting the Decision to Use Virtual Care

The providers described clinical factors that impact their decisions about whether to see a patient virtually or in person. The common clinical factors cited include patient acuity, a need for additional tests or labs, changes in the patient’s status and overall stability, and a visit with a patient who is new or has no record of recent medical examination.

#### Patient Acuity

The providers indicated that acute, newly emergent conditions proved most difficult to assess virtually. In particular, they noted that patients’ reports of pain were often challenging to assess virtually, since they were unable to physically examine sensitive areas to help in making a diagnosis. On the other hand, chronic conditions were better suited for virtual management, particularly if patients had already been diagnosed and had an established medical plan. Blood pressure and blood sugar management were characterized as 2 examples of chronic conditions that may be easily managed using virtual care. Elaborating on this observation, a spinal cord provider explained as follows:

Most of the time, you cannot [make a diagnosis] without laying hands on the patient. But [when it’s] just blood pressure management, blood sugar management, you don't have to have a patient face-to-face encounter. You can do only virtual.

#### Need for Additional Tests or Labs

Conditions that required lab draws or imaging to accurately diagnose were described as difficult to manage. The providers noted that patients who were able to have tests performed prior to their virtual visit were much more likely to have a productive visit, but because tests are often completed at the time of the in-person visit, previsit workups were reportedly uncommon. Hence, the providers noted that if a patient needed lab tests, additional virtual visits were often necessary to complete their assessments and ultimately make a diagnosis.

#### Changes in Health Status

The providers noted that patients who reported changes in clinical status and overall well-being were less appropriate for virtual care. These changes often signaled to providers the need for a comprehensive, in-person physical examination rather than a virtual exam. Some examples of health status changes that clinicians felt warranted an in-person visit included unexpected weight gain or weight loss and fluctuating or inconsistent symptoms accompanying a diagnosed chronic condition. For example, a cardiologist noted the following:

[If] I have a visit with a patient that’s either a telephone or a [video visit] and identify that there are some factors that are starting to concern me——in general it’s weight, shortness of breath, new symptoms that I wish I could have a physical exam or be able to examine the patient—then I will follow those telephone visits … with an in-person visit generally in the next couple of weeks and sometimes more urgently.

Conversely, the providers indicated that patients who reported a stable and consistent health status made for better candidates for virtual care.

#### New Patients and Individuals With No Recent Visit

Providers across all specialties maintained the view that first visits and new patients should be seen in person if possible. This view held steady despite the wide variety of conditions being assessed and treated among the providers who participated in the interviews. For example, a physician who treats patients with spinal cord injury stated the following:

In terms of pain, you have to have at least the first encounter in person, because you have to do a special test, you have to examine to see specificity, to palpate, to see joints, range of motion.

Additionally, patients who had not been seen in person for an extended period (2 or more years) were considered less ideal virtual care candidates. However, providers noted that they felt more comfortable offering virtual care when the patients had been recently seen by other providers within the medical system and for whom extensive notes were available.

### Patient Factors Impacting the Decision to Use Virtual Care

While the providers largely focused on the clinical needs and circumstances of patients when determining whether a virtual visit would be appropriate, they also described several patient-related factors that influenced decision-making, including a patient’s ability to articulate their symptoms and needs, ability to use the technology associated with virtual visits, general preferences for in-person visits, and access to a caregiver to assist with the virtual visit.

#### Ability to Articulate Symptoms or Needs

The providers explained that patients who were able to communicate their symptoms or needs in a robust and reliable way made for the best virtual visit candidates. Patients who had challenges describing their symptoms, difficulty recalling the timing of certain events or the onset of specific symptoms, or challenges describing physical changes or abnormalities left providers less confident in their virtual assessments. For instance, a primary care provider described how she imparts this advice to residents:

I tell the residents as we’re seeing patients, one of our first decisions to make is, “Can I safely continue this visit in this fashion, or is there no way I’m going to get enough data by history that I can end at a point where I feel like I’ve safely cared for the patient?”

The providers admitted that relying on patients’ accounts rather than their own hands-on assessments required a comfort level with virtual assessments, which often took time to develop. In response to this, the providers noted that they had to hone their history taking skills to feel confident with the information patients were relaying to them.

#### Availability and Accessibility of Technology

The providers indicated that patients needed both personal technology (eg, home computer, tablet, or smart phone) and reliable broadband access to participate in virtual visits. They described many instances of initiating virtual encounters with patients, only to discover that the video or sound quality was poor, and subsequently wasted valuable clinical time troubleshooting these technology-based problems with patients. In such cases, they would either try to follow up by telephone or simply reschedule in-person visits. The providers also noted that individuals with specific clinical or physical characteristics frequently had challenges with virtual visits (eg, older patients with cognitive disabilities such as dementia or patients who experienced sensory loss, namely hearing and visual impairments). A quote from a primary care provider illustrates this point:

Like hearing can become a huge problem. If hearing difficulties are too severe, it's really hard to have an appropriate visit. There's something with the tech, the video that I feel like people just can't hear you as well. I'm not sure if it's the delay and it throws off the mouth reading or something.

However, despite these challenges, the providers cautioned against assuming that all older patients or patients with sensory loss were inappropriate for virtual care, since they could think of many exceptions to this general observation.

#### Access to Caregiver Assistance

Finally, the providers noted that having another individual available to assist the patient, typically a caregiver or family member, increased their likelihood of conducting a virtual visit. Particularly among patients with mobility issues, sensory loss, or cognitive impairment, a caregiver was often able to help the veteran troubleshoot technology issues, assist with physical exam maneuvers, or help capture images providers needed to fully assess the patient. Underscoring this point, a palliative care provider explained how they suggest involving caregivers in assessing pain in areas that may be difficult for patients to reach:

I always ask the patient “Does it hurt to touch?” And if there's a spouse or another person or a family member or any other person there, I might ask them to touch it.

#### Preferences Regarding Virtual Visits

The providers reported that some patients preferred in-person visits to virtual visits and were therefore reluctant to engage through virtual care if an in-person visit could be conducted in a timely manner. They noted that some patients crave face-to-face interactions with their providers and report that the video format fails to replicate that connection. For others, this preference was also attributed to a lack of digital literacy skills and inadequate patient support to help facilitate their use. With additional instruction and digital familiarity, some of these patients could grow more accepting of virtual care.

Nevertheless, the providers speculated that patient’s preferences were unlikely to change and that they would continue to opt for in-person visits when given the choice. For instance, a primary care provider reflected as such:

My perception of my patients is they're not entirely comfortable never seeing me in person, especially new patients who I've never met. I think most of them feel like, “I'd like to meet you at some point.” I think that's always going to be a need there.

In these scenarios, the providers noted that they would often comply with patients’ preferences and opt to see the patient in person rather than virtually. The COVID-19 pandemic, however, necessitated at times that visits be virtual, even when patients preferred in-person care.

### Provider Factors Impacting the Decision to Use Virtual Care

While playing less of a role in real-time decision-making around virtual care, the providers also described how factors related to their own acceptance of and comfort with virtual care modalities impacted their decision-making. In addition, they noted that acquiring training on how to assess patients virtually would likely lessen their discomfort and encourage them to provide virtual care more often and to more patients with diverse clinical needs.

#### Comfort With and Acceptance of Virtual Technology

First, the providers argued that assessing patients virtually required a general acceptance of the format and a recognition that it necessitates a different approach to patient assessment and evaluation. While the providers in our sample largely appreciated virtual care, they described colleagues who lamented the shift to virtual care and found it challenging to adapt their clinical care to the new format. This acceptance provided a foundation for providers to improve their virtual diagnostic skills and increase the likelihood of engaging in a virtual visit with a patient. A primary care provider elaborated on this point as follows:

You have to accept the strengths and deficits of video [visits] and don't try to make it into a total replacement for a face-to-face visit, because if you're more comfortable listening to their symptoms, listening to what they tell you and they can relate to you pretty well how much edema they have and where it was before, and if you accept that, then you can get more done.

#### Virtual Physical Exam Skills

In most instances, the providers described learning to provide virtual care as a process of “just figuring it out,” while also drawing on the fundamentals of their clinical training. In this process, many acquired new skills and adopted new strategies for conducting virtual physical exams, including asking patients to engage in specific maneuvers or provide information not typically asked for in a face-to-face visit. The providers described how conducting virtual exams increased their awareness of the observations they make about patients and their physical health during in-person exams. Virtual exams required deliberate attention to those missing elements. For example, one primary care provider explained:

You're assessing the speed they're getting up and moving around, so you have to make sure to ask them to walk around. And so, I think that there is a potential to miss things if you haven't gone through the process of saying, okay, what are the things that I'm likely to miss as a provider given this particular modality, and then how can I try to counter those with just some things on your internal checklist that you want to make sure to ask about?

In this last example, asking patients to stand and walk around while on video was one way to assess gait and movement. The providers’ confidence in and acquisition of these skills increased the likelihood that they would opt to treat and assess a patient virtually.

## Discussion

### Principal Findings

In this qualitative study of VA providers, we found that a complex set of clinical, patient, and provider factors influences a provider’s decision about whether to provide care virtually or in person. Many of the providers in our study referenced scenarios where virtual visits had already been scheduled and initiated, but through examination of patients, they realized an in-person visit would be more clinically appropriate. Such instances added an additional visit for both patients and providers, contributing to potential waste and redundant services. This highlights the value for providers of knowing a priori which scenarios and which patients might be more appropriate for virtual care. Here, we detail several ways that these findings may be used to optimize the use of both virtual and in-person care.

First, the providers noted that, in many cases, a high-quality virtual visit requires some collection of information or data from the patient. As the providers have made the leap to virtual care, many have mourned the loss of data that would be more easily accessible in a traditional in-person visit, such as vital signs and physical exam findings [18]. Some providers have found solutions in home devices such as blood pressure cuffs, blood glucose monitors, pulse oximeters, and scales, all of which can help them to form a more complete picture of patients’ vital signs and other important information for decision-making [19,20]. The increasing availability of wearable and other patient-facing digital technologies, including exercise monitors (eg, FitBits and Apple Watches), smartphone-associated portable electrocardiograms, and home-based lab testing may offer additional opportunities to collect key information outside the in-person visit, although there is still a need for evidence about the reliability and consistency of data in different circumstances [21,22]. Augmenting a virtual visit with these technologies may mitigate the risks that the providers in our study noted when they must rely on imperfect or incomplete patient-provided histories.

Second, specific skills and training are required to conduct effective virtual visits and spare providers from “figuring it out as [they] go.” Several efforts are underway to develop and disseminate training and instruction on virtual care and to integrate these domains into standard medical school and residency curricula [7,23]. Additional training resources should target mid- or later-career clinicians, since they are less likely to be exposed to interventions geared toward medical trainees. The American Medical Association as well as other organizations and societies have developed resources to help clinicians build telemedicine physical exam skills [24] and communication skills such as “digital empathy” [25]. These resources include guidebooks as well as informational webinar series and videos. Others have created helpful guides for conducting patient-assisted physical exams [26].

Third, there is a need for guidelines to help determine whether a specific visit should be scheduled in person or virtually. The providers largely maintained that patients without recent visits or presenting with new or higher acuity problems might not be best served by virtual visits. Both for building patient rapport and for ensuring a more complete mental model of a patient’s condition, it may well be best for an initial patient visit to occur in person for most patients and clinical situations [25]; some have suggested newly diagnosed patients should always be seen in person, at least initially, until medication regimens can be safely established [27]. Nevertheless, there is mounting evidence that with proper training and protocols in place, even high-acuity clinical circumstances can be safely assessed virtually and may even decrease overall rates of emergency care use. For example, Wray et al [28] demonstrated how a “tele-urgent care program” that provided care for a variety of clinical scenarios was safe, effective, and led to the decreased use of emergency departments. Such findings may provide further confidence to virtual care providers that virtual care can provide safe access to care in a variety of clinical scenarios.

Finally, attention to equity is needed to ensure that all patients have opportunities to build digital literacy skills [29], have access to the technology and receive the support they need to participate in virtual visits. Failure to attend to these issues may contribute to further inequity in health care provision and outcomes [30]. Health care organizations have attempted to respond to this digital divide in a variety of ways. For instance, the VA initiated a tablet distribution program, in which at-risk, high-need patients are provided with video-enabled tablets equipped with internet service. This program has resulted in improved access and continuity of care, with high satisfaction rates among Veterans [31,32]. In addition, studies have found that providing patients with hands-on instruction on how to use new technologies may further ameliorate a lack of digital literacy skills [33]. Moreover, functional limitations (eg, loss of eyesight and hearing as well as dementia) also created barriers to patients’ use of virtual care in this study. Incorporating principles of universal design, which advocates for designing products and services that can be used by all individuals to the greatest extent possible, may abet some of these issues and ensure accessibility for all patients [30,34,35].

Even with the array of tools and strategies described above, it is unlikely that all combinations of providers, patients, and clinical scenarios will ultimately prove ideal for virtual care. Given the dramatic expansion of virtual care since the onset of the COVID-19 pandemic, the existence of virtual care is effectively a foregone conclusion; what is essential to uncover at this stage is how and when to best use the various visit modalities at provider and patients’ disposal. The observations providers shared in this study are useful for generating hypotheses on how to integrate virtual and in-person care.

### Limitations

This study has several limitations. First, the sample was limited to providers at the VA; thus, provider experiences may not apply to other settings, particularly those with different reimbursement models. Fee-for-service systems, the predominant mode of health care delivery in the United States, may reimburse virtual visits differently from in-person visits and pose additional incentives or disincentives to use virtual care. However, we were able to assess a broad array of providers across a variety of geographic regions, improving the transferability of our findings. Second, this qualitative study about provider perceptions does not assess the impact of these factors on quality of care and patient outcomes, which would provide value in discussions about the degree to which these criteria should inform guidelines and protocols. For example, while the providers noted that remote monitoring devices and other technology increased their comfort and confidence in virtual examination, the actual impact of this factor on quality and safety of care warrants further evaluation. A final and perhaps most significant limitation is that this study does not assess decision-making in real time and instead relies on providers’ reflections on their decisions, an inherent limitation of qualitative interviews. Though challenging to carry out, direct observations of clinical practices may offer a more realistic account of provider decision-making around virtual care.

### Conclusion

This qualitative study found that providers within the VA consider a complex array of factors when deciding whether to offer or continue with a virtual visit. Clinical factors were the most dominant, but patient and provider factors also influenced the decision process. These findings can inform health system policies to ensure accessible, high-quality care, as well as policy maker considerations when adjudicating reimbursement levels for virtual care visits. Further development of tools, resources, and guidelines is needed to facilitate real-time provider decision-making about when to offer a patient virtual care.

## Abbreviations

VA: US Department of Veterans Affairs
